# An Artificial Intelligence Approach to Regulating Systemic Risk

**DOI:** 10.3389/frai.2019.00007

**Published:** 2019-05-29

**Authors:** Sharyn O'Halloran, Nikolai Nowaczyk

**Affiliations:** ^1^Columbia University, New York, NY, United States; ^2^School of International and Public Affairs, Department of Political Science, Quaternion Risk Management, Dublin, Ireland

**Keywords:** artificial intelligence, graph theoretic models, data science, machine learning, stochastic Linear Gauss-Markov model, financial risk analytics, systemic risk, financial regulation

## Abstract

We apply an artificial intelligence approach to simulate the impact of financial market regulations on systemic risk—a topic vigorously discussed since the financial crash of 2007–09. Experts often disagree on the efficacy of these regulations to avert another market collapse, such as the collateralization of interbank (counterparty) derivatives trades to mitigate systemic risk. A limiting factor is the availability of proprietary bank trading data. Even if this hurdle could be overcome, however, analyses would still be hampered by segmented financial markets where banks trade under different regulatory systems. We therefore adapt a simulation technology, combining advances in graph theoretic models and machine learning to randomly generate entire financial systems derived from realistic distributions of bank trading data. We then compute counterparty credit risk under various scenarios to evaluate and predict the impact of financial regulations at all levels—from a single trade to individual banks to systemic risk. We find that under various stress testing scenarios collateralization reduces the costs of resolving a financial system, yet it does not change the distribution of those costs and can have adverse effects on individual participants in extreme situations. Moreover, the concentration of credit risk does not necessarily correlate monotonically with systemic risk. While the analysis focuses on counterparty credit risk, the method generalizes to other risks and metrics in a straightforward manner.

## 1. Frontiers of Artificial Intelligence

Predicting the next financial crisis is like forecasting the weather, a plethora of variables must converge at just the right moment in just the right way, invariably, leading experts to arrive at wildly conflicting prognostications. Advances in artificial intelligence (AI) methodologies have enhanced the robustness of such predictive models by introducing schemes based on skeletonization that extract vertices and edges from an initial graph and algorithms that prune unlikely outcomes by sifting through hundreds of thousands of factors to match shapes to known prototypes^1^.

Artificial intelligence, which incorporates machine learning and data science, places data within a context through pattern recognition and iterative learning. What is new about the latest incarnation of the AI framework is that its draws on many disciplines, such as statistics and computer science, but also biology, psychology, and game theory, and employs a myriad of techniques, including:

1. Rules-based systems that set parameters and conditions to enable scenario testing;
2. Machine learning that applies algorithms to decipher patterns and linkages in the data by continuously updating “learning” through an iterative process;
3. Neural networks that identify interconnected nodes through multi-layered data to derive meaning;
4. Deep learning that leverages pools of high-dimensional data to identify patterns of patterns; and
5. Pattern recognition that uses tools, such as natural language processing to classify and interpret data.

What does this methodology tell us about predicting financial disasters or, even more importantly, how to avoid them? The turmoil following the 2008 collapse of Lehman Brothers, gave rise to a lively debate on how to regulate financial markets. Governments have imposed a number of regulations to reduce systemic risk or the possibility that an adverse event at a single financial institution could trigger severe instability or the collapse of an entire industry or economy. To mitigate effects of cascading defaults, for instance, regulators introduced the collateralization of derivative trades and incentivized dealers to clear trades on centralized exchanges as opposed to over-the-counter.

The financial crisis not only called into question the soundness of such regulations, but also the process to evaluate the efficacy of new regulations being put into place. Although a decade has passed, regulators and industry participants alike failed to arrive at a consensus on: (1) Have the regulations implemented post-crisis reduced systemic risk? (2) How can we predict the impact of a financial regulation before it is implemented? and (3) How can we evaluate which regulation is best to avert yet another “Financial Katrina?” As many governments once again face pressure to rollback far reaching financial legislation, it is necessary to know which regulations promote safety and soundness of the financial system and which add undue burdens on markets.

In this paper, we analyze credit exposures created by contracts among financial institutions that arise when one party defaults or fails to repay the contracted amount, or counterparty credit risk. We develop a graph model that characterizes a financial system as a network, similar to skeletal representations in meteorology, where the nodes of the graph represent a bank and the vertices represent credit relations, each with various weights. We introduce an analytic tool that simulates a financial system based on real case trade data. Through an iterative process, we evaluate, predict and optimize the amount of collateralization required to mitigate counterparty credit risk at the trade, bank and systemic level.

The analysis shows that collateralization reduces the costs of resolving risk in a financial system, yet it does not change the distribution of those costs among banks and can have adverse effects on individual participants in extreme situations. Consistent with the work of Battiston et al. (2012a,b) we also find that diversification is not sufficient to ward against systemic financial failures; indeed, it may exacerbate it. The analysis measures the impact of collateralization on counterparty credit risk exposure in the derivatives market, but the method generalizes to other types of risks and metrics in a straightforward manner. The approach developed enables regulators and industry participants alike to conduct iterative scenario testing and thereby provides a unique opportunity to make informed decisions about the impact of public policy before the next crisis strikes.

## 2. Models in Crisis: A New Approach

The 2008 financial crisis was the perfect storm of failures: Wall Street, regulators, hedge funds, all played a part. Government's response has been to introduce a number of new regulations to improve the safety and soundness of the banking system as well as mitigate systemic risk. These include: capital buffers, leverage requirements and restrictions on derivatives. This has taken place at both domestics and global levels.

The question is, given all these regulations are we better off now than before? In particular, is the financial system more transparent and accountable than prior to the crisis? After all it was the oblique, complex derivatives that exasperated the mortgage crisis and almost brought down the international system in the first place.

The financial industry's response to these regulations has been to build black box risk models developed, for the most part, in institutional silos. The implication is that financial firms currently conduct risk exposure analysis absent shared standard models to use as benchmarks and validate results.

Yet, regulations require transparency and flexibility, and these requirements cannot be met by traditional silo-ed approaches. In response, collaborative efforts among academia, industry, and government have formed. Even the banks have come together in a previously unheard of data consortium, AcadiaSoft.

This reorganization has been accompanied by paradigm shifts from proprietary, homegrown software to open source. Even in financial risk management open source solutions, such as ORE, see Open Source Risk Engine (2016), have emerged. This trend has facilitated the use of AI technologies in the solution space, including: machine learning, natural language processing, AI and neural networks, provide powerful tools to augment risk analysis. In addition, these technologies provide new ways of developing models.

### 2.1. Open Source Risk Engine (ORE)

**Figure 1:**
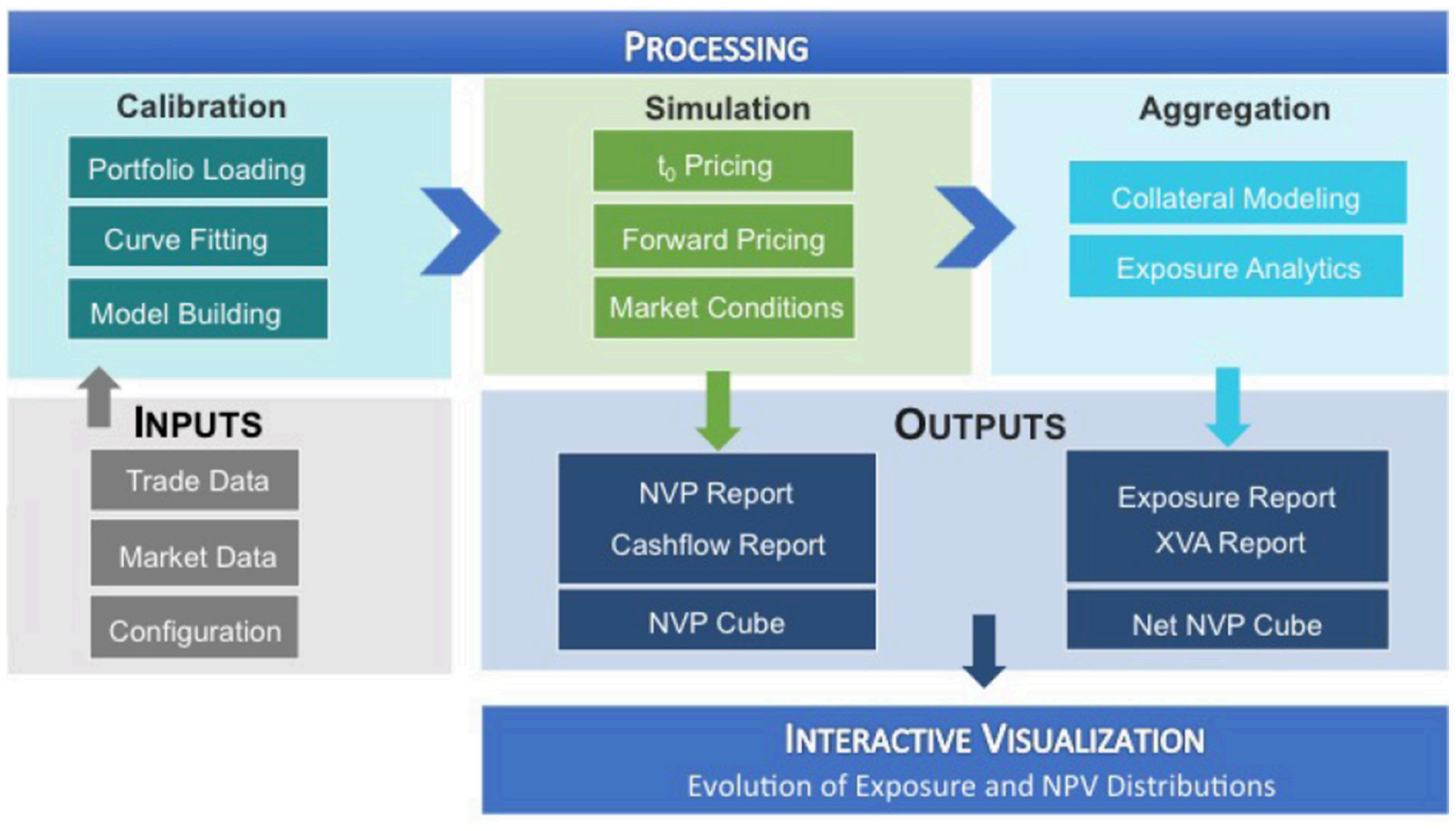
Open source risk engine (Reprinted with permission by Columbia University Press).

ORE computes the risks in a derivative portfolio from the perspective of a single bank. Schematically, it works as follows, see also Figure 1: It consumes trade data, market data and some configuration files as inputs, identifies all risk factors of the trade portfolio and performs a MonteCarlo simulation. This allows the computation of risk analytics at portfolio, asset class, and counterparty levels. See Lichters et al. (2015); Open Source Risk Engine User Guide (2017).

These analytics provide a benchmark that can be shared by regulators and industry participants to calibrate models around risk tolerance. As the assumptions are commonly known, it enables conversations around why and how various models deviate from the standard benchmarks.

### 2.2. A Systemic Risk Engine

One can aggregate firm specific risk metrics produced by the ORE into a systemic risk engine to assess the impact that regulations have on the financial system as a whole. This requires that the analysis takes into account not only the impact that financial transactions have on a financial institution but also the impact that each institution has on the system. Netting these input and output effects provide a more realistic picture of the impact of a regulation on the risks in the financial system. Moreover, adopting graph modeling enables visualization, calculation and testing of the robustness of various hypotheses under alternative parameter assumptions. More technical details on the technology stack used in the simulation can be found in Anfuso et al. (2017); O'Halloran et al. (2017b).

### 2.3. Columbia Data Science Institute FinTech Lab

The Columbia FinTech Lab housed in the Data Science Institute provides an easily accessible demonstration of how these tools can produce risk analytic measures. The Fintech Lab website, see Columbia University Fintech Lab (2018), provides a graphic display and interface that demonstrates how such analysis can be conducted.

## 3. Use Case: Systemic Financial Risk

ORE has been built to compute the risks in a derivatives portfolio from the perspective of a single bank with purposes of serving as a bank risk management system or validating such a system. Its applications have an interesting pivot, however. Because, the computations of those risks from the perspective of one bank requires the above mentioned inputs, market data, trade data, netting agreements and other simulation parameters, one can use ORE to compute systemic risk, by running the computation from the perspective of all banks in a system.

The results include all risks of all banks in a financial system. As the same models are used for each bank, the resulting risk metrics are consistent and comparable across all banks. Those metrics can be computed under different regulatory regimes, allowing a consistent evaluation of the impact of financial regulation on systemic risk.

In practice, performing such a computation is difficult as one crucial input, the trade data of all the banks in the system, is proprietary and thus inaccessible. However, if the purpose of such a computation is to evaluate the impact of a financial regulation in general or to guide regulatory decision making bodies, it is, in fact, undesirable for the outcome to depend overly on current trade data. Trading activity in the global financial system is significant. Millions of transactions change the trade portfolios of the market participants every day, even every second. Changes in financial regulation, however, happen over a period of decades. The regulations around Initial Margin, for instance, a direct reaction to the financial crisis in 2007–2008, are still not fully implemented and will not be implemented fully before the early 2020s. Given the different time scales for changes in trade portfolios and changes in financial regulation, it would be an undesirable feature of financial regulation if its impact strongly depended on current trade data as this would signal overfitting of regulation to the current market.

Ideally, financial regulation should have the desired impact and that impact should be largely invariant under trading activity. Consequently, the evaluation of a regulation should be largely independent of changes in trade data. The precise trade data of the current financial system, therefore, should not be needed to evaluate the impact of a regulation. What is needed to study the impact of a regulation on a financial system is simply trade data, preferably as realistic as possible, but not necessarily the live deals of the current dealer banks. Our approach is to use a simulation technology. We randomly generate entire financial systems, including trade data, and calibrate those random generators to realistic distributions. The result is a representative sample of possible financial systems, which is transparent and completely accessible on all levels, from a single trade to the entire system.

### 3.1. Literature Review of Systemic Risk Metrics

**Figure 2:**
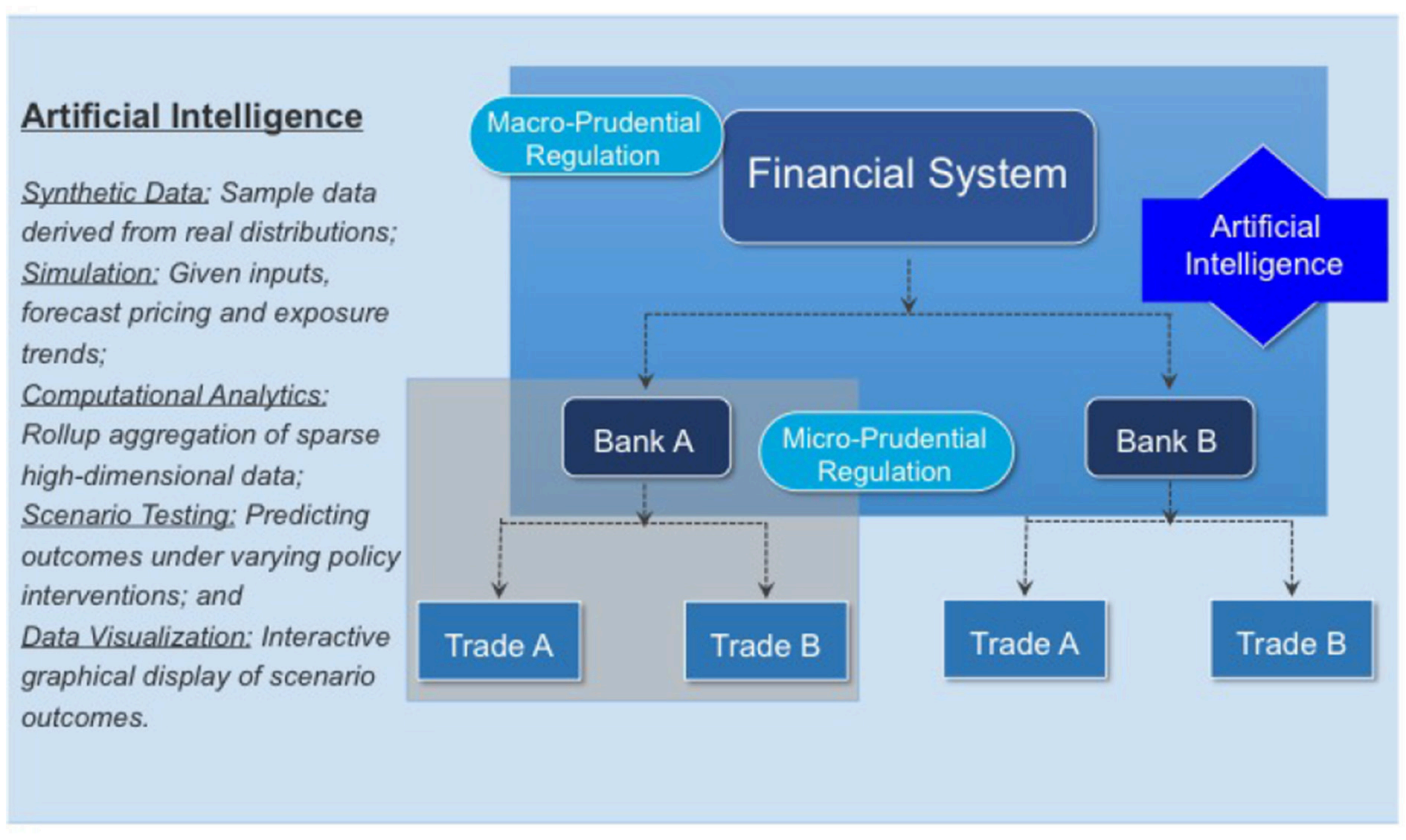
Using artificial intelligence to close the gap between micro- and macro-prudential regulation (Reprinted with permission by Columbia University Press).

This simulation approach has the advantage of bridging the gap that traditionally separates micro- and macro-prudential regulation, see Figure 2. The micro-prudential side considers a single bank in all its complexity and is primarily interested in the risks this bank is exposed to as a result of the trades in its portfolio. The metrics in which those risks are measured are standardized and their use is enforced globally by regulators. Examples include Value-at-Risk (VaR) to measure market risk, Effectivized Expected Positive Exposure (EEPE) for credit risk, Liquidity Coverage Ratio (LCR) for liquidity risk or a Basel-II traffic light test for model risk. Even though the concrete value of a metric like EEPE can differ between two banks that use internal models, the regulatory framework around internal models is designed to minimize those differences and the method, at least, is consistent. The only drawback of the micro-prudential view is that it considers only one bank in isolation making it difficult to study systemic risk.

In contrast, macro-prudential regulation considers an entire financial system with all its banks, but evaluates each and every bank from a high level perspective only. From a macro-prudential view, the amount of risk a bank is exposed to is of less interest than the amount of risk a bank induces into the financial system. In particular, the question on whether or not a bank default could result in the default of the system is of particular importance (“too big to fail”). An excellent overview is provided by Battiston and Martinez-Jaramillo (2018) of the relationship between micro-prudential policies, which focus on individual exposures and leverage and capital ratios, and macro-prudential network-based policies.

In sharp contrast to the micro-prudential risk metrics, there is no clear definition on what systemic risk precisely means nor how it should be measured. In Bisias et al. (2012), the U.S. Office for Financial Research discusses 31 different metrics of systemic risk^2^. A closer look at these metrics, however, reveals that these are not simply different mathematical functions measuring the same quantity, but different underlying notions of systemic financial risk. Most of these metrics focus on the analysis of market data, such as housing prices or government bonds and their correlations. For instance, Billio et al. (2012) use Principal Components Analysis (PCA) and Granger Causality to study the correlations between the returns of banks, asset managers and insurance. Unfortunately, most of those macro-prudential metrics are unsuited to guiding decision making bodies or regulatory interventions—precisely because their micro-prudential nature remains unclear (with CoVaR, which relies on a quantile of correlated asset losses, being a notable exception; see Adrian and Brunnermeier, 2016).

More recently, Sedunov (2016) compares the performance of three institution-level systemic risk exposures to forecast the financial crisis, including Exposure CoVar, Granger causality, and Systemic Expected Shortfall. Using data from the 25 largest U.S. banks, insurers, and brokers, the analysis shows that CoVar is the measure that best forecasts the within-crisis performance of financial institutions over multiple crisis periods. By contrast, neither Granger causality nor expected shortfall metrics predict within crisis performance. A key indicator in forecasting crisis exposures is the size of the financial institution.

### 3.2. AI: Bridging the Gap Between Micro- and Macro-prudential Regulation

As Figure 2 demonstrates micro-prudential regulation is directed toward the safety and soundness of an individual bank. Financial crises, however, result from the external actions of a bank, which may or may not be correlated with its compliance with regulatory standards. A lessons of the 2007–09 crisis is that macro-prudential regulation focused only on the risks taken by individual banks is insufficient to prevent crises.

An AI framework provides a way to bridge this gap. First, synthetic data of a financial system can be derived by sampling data from real market, portfolio and bank trades. Second, given these inputs, simulations can be constructed to forecast pricing and exposure trends. Computational analytics provide models for prediction and accuracy testing of sparse, high dimensional data. Scenario testing enables comparisons of different policy interventions on market outcomes. Finally, graphical visualization based on pattern recognition facilitates classifying outcomes.

### 3.3. Weighted Degree Metrics

This 2-fold divergence in metrics—the gap between micro- and macro- prudential regulation and the different notions of systemic risk—is unfortunate from a methodological point of view. The various notions of systemic risk are a consequence of the fact that this is a relatively new field and that the financial system and hence systemic risks are very complex and have many different facets. The gap between micro- and macro-prudential regulation has historic origins: The obvious approach of studying the macro-prudential impact of a regulation on an entire financial system as an aggregation of all its micro-prudential impacts has failed in the past due to the complexities of both levels.

In recent years there have been tremendous technological advances in handling big and highly complex data sets. Therefore, our approach is to use the standardized micro-prudential risk metrics and aggregate them in a graph model of systemic risk.

The advantages of this methodology are manifold. First, of the 266 papers reviewed by Silva et al. (2017), the analysis shows that only 20 articles used a combination of computational, simulation, and mathematical modeling. AI techniques enable iterative hypothesis testing to decipher patterns and linkages in the data, thereby providing more robust models and estimates of systemic risk. Second, Battiston and Martinez-Jaramillo (2018) note that existing research addresses systemic risk from either a micro-prudential or a macro-prudential level, absent any analysis of how link the two. By contrast, we derive a systemic risk metric from the ground up. The total risk exposure in the financial system is an aggregate estimate of individual firms' credit risk exposure, thereby providing an indicator of how much risk a firm generates and how much it absorbs. And third, as documented by Silva et al. (2017), network analysis (Battiston et al., 2012a,b), cascade models (Capponi and Chen, 2015), and even examinations of the topological structure of inter-bank networks (Caccioli et al., 2015) are readily adopted constructs to evaluate contagion effects among financial institutions. Here, we employ the mathematics of graph models to analyze the credit risk in financial systems.

## 4. Graph Model of Systemic Risk

**Figure 3:**
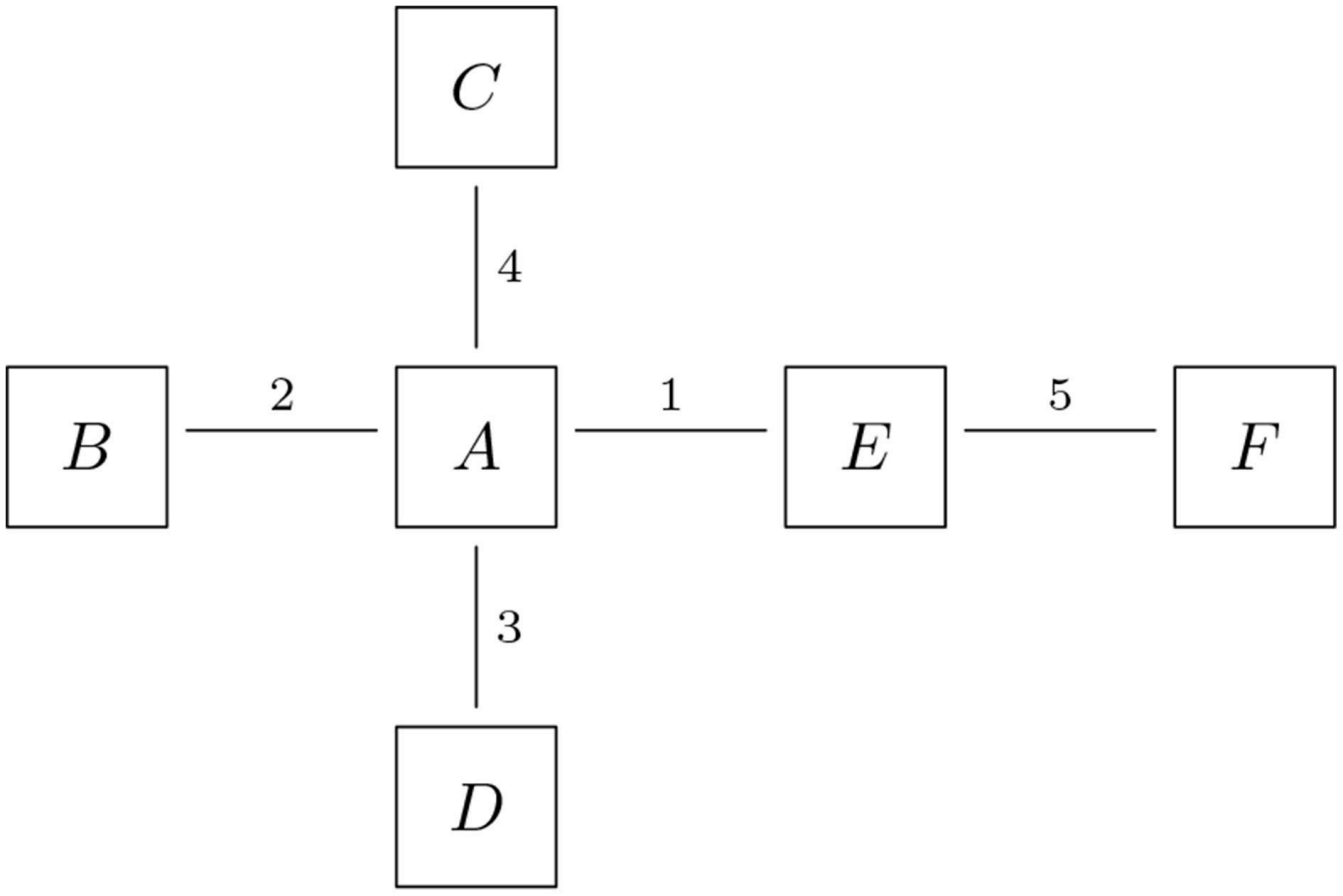
Trade relations: the nodes represent the banks, the links represent the trade relations and the labels on the links represent the trade or portfolio IDs (Reprinted with permission by Columbia University Press).

The trade data in a financial system is naturally organized in an undirected *trade relation graph* *G* = (*B, T*): The nodes *B* represent the banks and the links *T* represent the trade relations. The graph is undirected because a trade relation is symmetric—a deal is only a done deal if both sides sign it. For formal details on graph models, see Erdős and Rényi (1959, 1960); Bales and Johnson (2006). An example of a trade relation graph is shown in Figure 3, where six banks (labeled *A*-*F* here) are trading bilaterally with each other in five trade relations. Any additional data on the trade portfolios can be attached to the links, for instance as a list of trade ids. The details of the trades are then stored in a database. This model serves both as a representation of a financial system and as a data format for the random generation of financial system, c.f. section 5.2. Optionally, one can also attach more information on the nodes in that graph, for instance a bank's core capital ratio.

**Figure 4:**
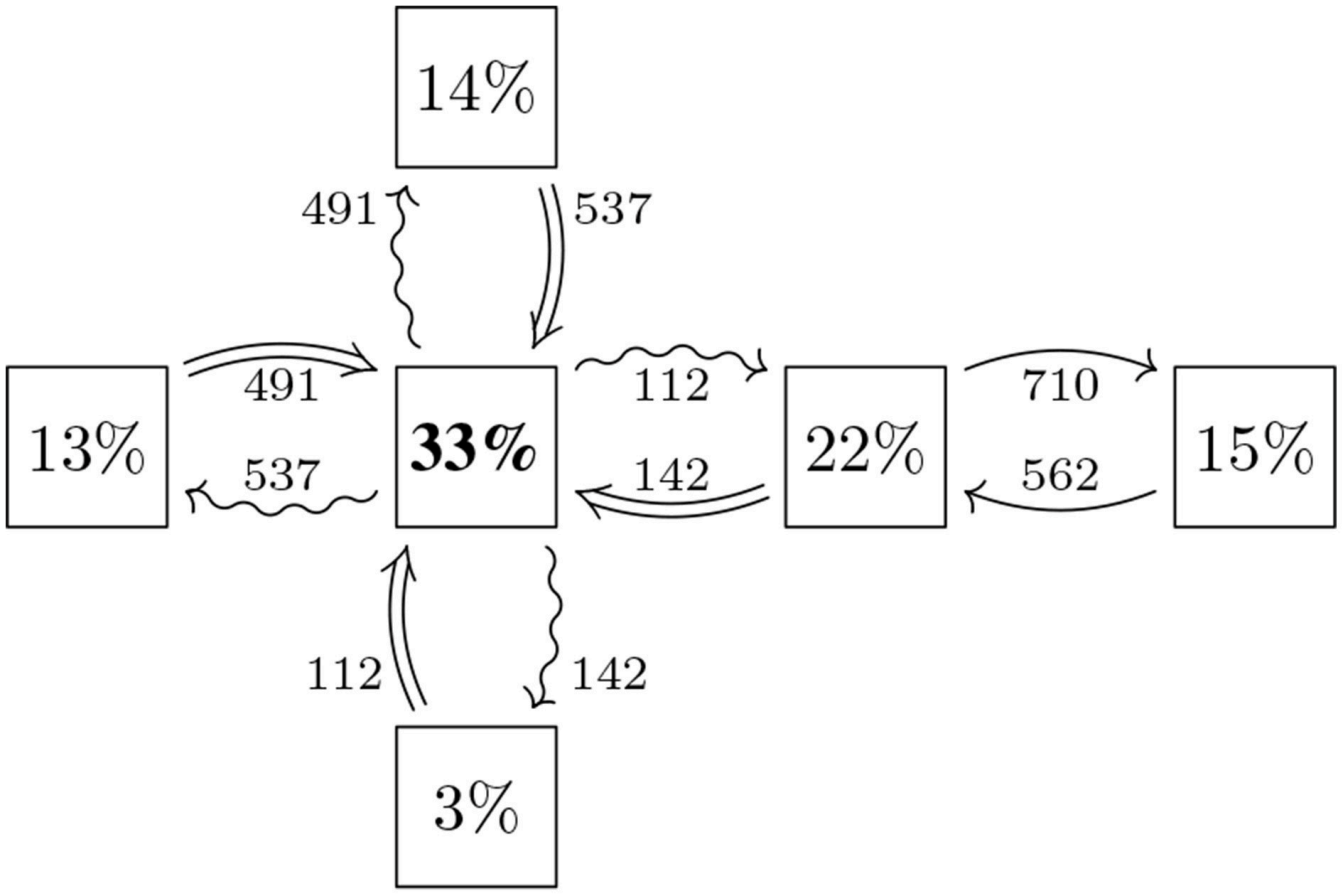
Exposures: the nodes represent the same banks as in Figure 3, the arrows represent that risk is induced from the bank on the tail onto the bank on the head, the weights on the arrows quantify that risk and the percentages in the nodes represent the share of risk induced by that bank (Reprinted with permission by Columbia University Press).

Each trade in a trade relation imposes various types of risks (as well as rewards) on potentially both banks and these risks can be computed in various metrics by means of mathematical finance. By computing a fixed set of risk metrics for all trade relations in a trade relation graph, we obtain a risk graph that captures the risks between all the various banks in the system, see Figure 4 for the example. Formally, the risk graph RG = (*B, A, w*) is computed out of the trade relation graph as follows: The risk graph has the exact same nodes *B* as the trade relation graph, but each undirected trade relation *t* ∈ *T* is replaced by two directed arrows *a*_1_, *a*_2_ ∈ *A* representing the risks the bank at the tail induces onto the bank on the head and vice versa as a consequence of their trade relation. Finally, we attach a (possibly multivariate) weight function *w*(*a*) onto the arrows *a* ∈ *A* that quantify the risks. An example we will use later is EEPE (Effectivized Expected Positive Exposure) to measure credit risk^3^. Another example could be the PFE (Potential Future Exposure) over a certain time horizon at a fixed quantile (analogous to US stress testing). Notice that the amount of risk that is induced by a bank *b*_1_ onto a bank *b*_2_ may or may not be the same as the amount of risk induced from *b*_2_ onto *b*_1_ even though both are in the same trade relation. For example, the loss an issuer of an FX option might suffer as a result of the buyer defaulting is at most zero, while the buyer can in theory suffer a unlimited losses. Notice that this use of a directed graph to model exposures in a financial system is consistent with (Detering et al., 2016), who use this to study default contagion.

The weight functions, that is, the risk metrics, can be computed using ORE. The resulting data produces a weight *w*(*a*) for each arrow *a* ∈ *A* in a risk graph. This provides a complete picture of risk in the financial system modeled by the trade relation graph in established micro-prudential risk metrics. We then aggregate this data by a purely graph theoretic construction from the arrows of the risk graph to the nodes and then further to a systemic level as follows: For each bank *b* ∈ *B*, we compute the weighted in/out-degree

(1) $$\begin{array}{l} w^{-}\left(b\right):=\underset{\begin{array}{c} a\in A\\ a\text{ ends at }b \end{array}}{\sum }w\left(a\right),\;w^{+}\left(b\right):=\underset{\begin{array}{c} a\in A\\ a\text{ starts at }b \end{array}}{\sum }w\left(a\right). \end{array}$$

The in-degree *w*^−^(*b*) represents the total amount of risk the bank *b* is exposed to from the system and thus corresponds to the micro-prudential view of *b*. The out-degree *w*^+^(*b*) represents the total amount of risk the bank *b* induces into the system and thus corresponds to the macro-prudential view of *b*. Therefore, this graph theoretic construction bridges the gap between the micro- and the macro-prudential by providing a coherent metric of both in the same model. In the example shown in Figure 4, the in-degree of the big bank *A* in the middle is *w*^−^(*A*) = 537+142+112+491 = 1282 and the relevant arrows going into *A* are highlighted as ⇒. The out-degree is *w*^+^(*A*) = 491+112+142+537 = 1282 and the outgoing arrows are highlighted as ↝.

In a second step, we aggregate the risk metrics to a system wide level by computing $w\left(G\right):=\underset{a\in A}{\sum }w\left(a\right)$ the total weight in the system. It is instructive to express the weighted in- and out-degree as a percentage of that total, i.e., to compute

(2) $$\begin{array}{ll} \rho^{-}\left(b\right):=\frac{w^{-}\left(b\right)}{w\left(G\right)}, & \rho^{+}\left(b\right):=\frac{w^{+}\left(b\right)}{w\left(G\right)}, \end{array}$$

a relative version of the weighted in/out-degree. In the example shown in Figure 4, the total amount of risk in the system in *w*(*G*) = 3, 836 and e.g., counterparty A has ρ^+^(*A*) = *w*^+^(*A*)/*w*(*G*) = 1, 282/3, 836 = 33%. Any of the quantities

(3) $$\begin{array}{l} w\left(G\right),\;\underset{b\in B}{\max}w^{+}\left(b\right),\;\underset{b\in B}{\max}\rho^{+}\left(b\right) \end{array}$$

are (possibly ℝ^*k*^ valued) metrics that capture the total amount of weight in the graph and its concentration. These metrics serve as weighted degree metrics of systemic risk.

## 5. Collateralization

The financial crisis exposed vividly the credit risk component in derivative contracts. Any two banks that enter into a derivative contract fix the terms and conditions of the contract at inception and both commit to payments according to the contract until it matures. While the rules on how to compute the payment amounts are fixed at inceptions, the payment amounts themselves are not as they depend on future market conditions. In particular in the interest rate derivatives market that has an estimated total aggregated notional in the hundreds of trillions, the maturities of these contracts can be several decades. This exposes the two trading counterparties to each others credit risk: A payment in 10 years would simply not happen if one of the counterparties defaults in 9 years. As a derivative contract with a defaulted counterparty is worth zero, a default induces a significant shock to the value of a derivatives book of a bank.

**Figure 5:**
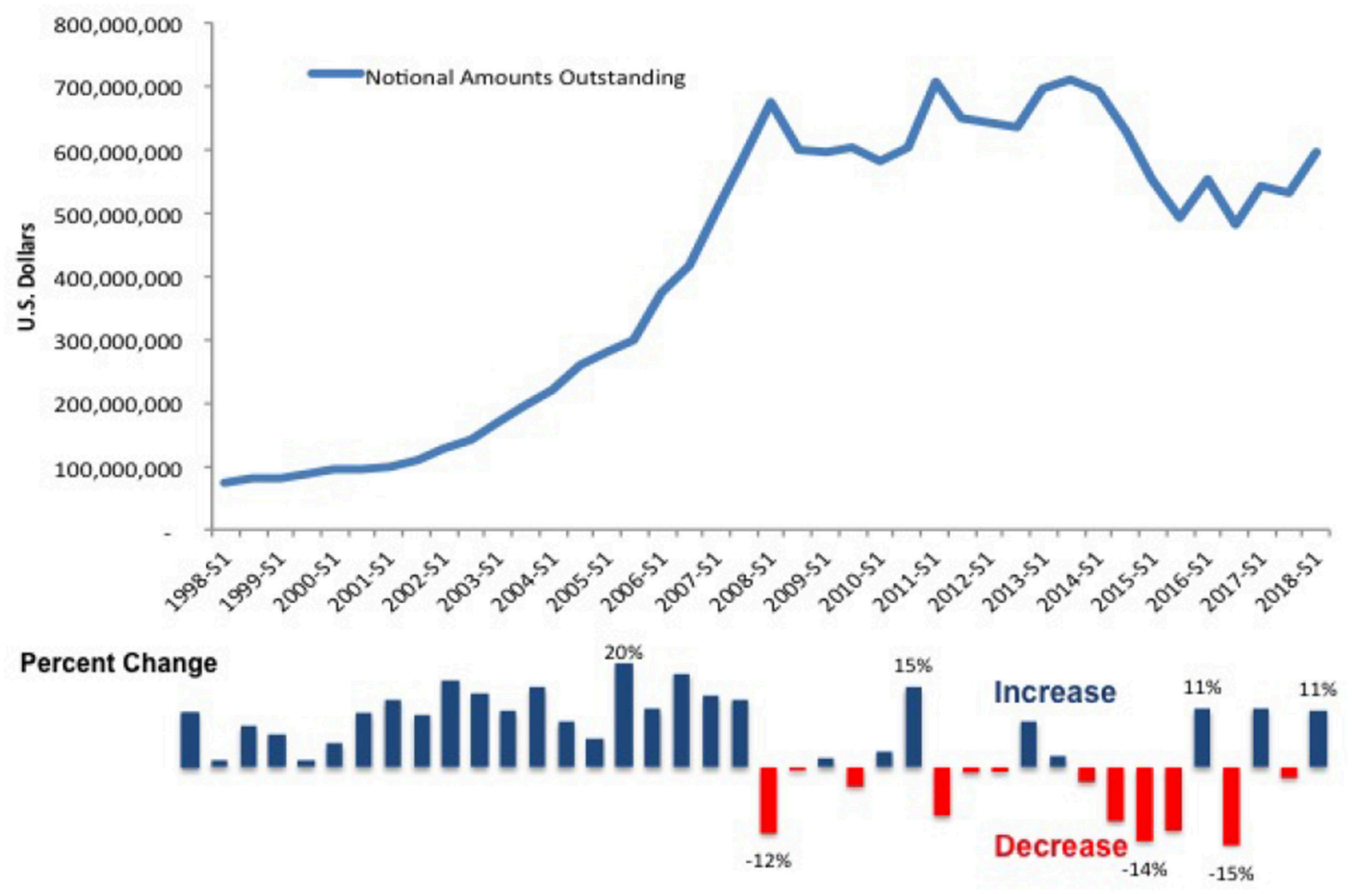
Notional value of over-the-counter derivatives, 1998–2017. See stats.bis.org; BIS derivatives statistics, OTC derivatives outstanding for all counterparties and risk categories on a net-net basis.

Figure 5 shows the magnitude of the over-the-counter derivative market. The top part of the chart displays the notional amounts of outstanding derivatives in millions of U.S. dollars from 1998 to 2018. The data covers all derivative types, e.g., currency and interest rate swaps, for all risk types and all countries. The graph illustrates a steeply rising trend that peaks during the financial crisis, 2007–2009. The bottom half of the chart shows the increases and decreases in the trend line. The onset of the liquidity crisis in the U.S. and the sovereign debt crisis in Europe led to decreases in derivative trading activity. The subsequent introduction of new regulatory standards to force dealers to trade derivatives through central counterparties (CCPs) or exchanges precipitated sharp declines in notional amounts. By the end-June 2018, however, the notional value outstanding had once again reached 595 trillion USD, close to pre-crisis levels. The resumption of an upward trend suggests that despite new regulations to push more dealers onto central clearing platforms, banks continue to use non-standard derivative contracts.

**Figure 6:**
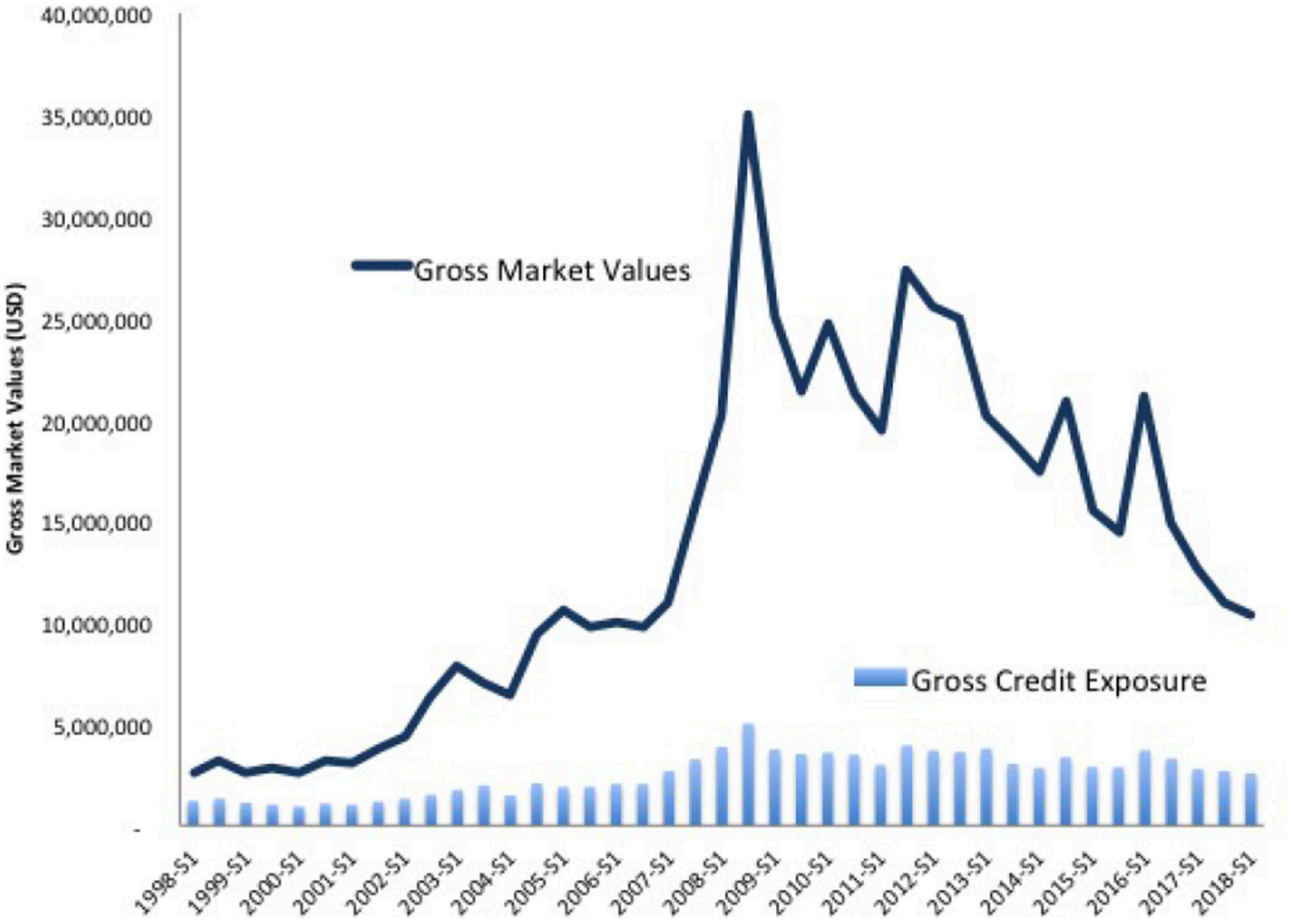
Gross credit exposure and gross market values, 1998–2018. See stats.bis.org; BIS derivatives statistics, OTC derivatives outstanding for all counterparties and risk categories on a net-net basis.

Figure 6 compares OTC derivative gross market values and gross credit exposure from 1998 to 2018. The solid line shows the gross values, which measure a bank's total exposure to financial markets or the investment amount at risk. Once again, the trend peaks before the crisis and declines afterwards. This time, however, the line continues its decent. For regulators, this indicates the success of stringent clearing and collateral requirements. By contrast, gross credit exposures, shown on the bottom of Figure 6 by the light blue bar chart, tell a different story. Credit exposure is the total amount of credit made available to a borrower by a lender and calculates the extent to which a lender is exposed to the risk of loss in the event of the borrower's default. The chart shows that while market values have decreased, credit exposures have remained unchanged. In short, the credit risk resulting from a failure has not altered even as the total amount of market risk has declined. Moreover, the proportion of outstanding OTC derivatives that dealers cleared through CCPs held steady, at around 76 percent for interest rate derivatives and 54 percent for credit default swaps (CDS)^4^.

These data highlight that regulatory interventions may have unintended consequences. Adopting an AI framework—e.g., generating synthetic data from real bank distribution, simulating a financial system, and conduct scenario testing by introducing policy interventions and compare outcomes, may help avert implementing poorly tailored policies.

For example, a standard financial regulation to mitigate credit risk exposures is collateralization. That means that the two counterparties exchange collateral (typically in cash or liquid bonds) with each other during the lifetime of the trade. In a first step, counterparties exchange variation margin (VM) to cover the current exposure to daily changes in the value of a derivatives portfolio, sometimes subject to thresholds and minimum transfer amounts. This regulation is already fully phased in. In a second step, on can post initial margin (IM) to each other to cover for the potential exposure to close out risk after a default would occur. A more detailed description of these regulations can be found in (O'Halloran et al., 2017a, section 4); see also the Basel Committee on Banking Supervision (2015); Andersen et al. (2016, 2017); ISDA (2016); Caspers et al. (2017).

### 5.1. Collateralization Regimes

These collateralization regulations lead to four different regulatory regimes:

1. All derivative trades are uncollateralized.
2. All derivative trades are VM collateralized, but some may only be partially collateralized due to thresholds and minimum transfer amounts.
3. All derivative trades are fully VM collateralized.
4. All derivative trades are fully VM collateralized and also fully IM collateralized.

For reasons of clarity, we exclude regime (2) from the present discussion. It is obvious that collateralization mitigates the exposure to credit risk on a micro-prudential level from the perspective of each counterparty^5^. We now test the hypothesis that collateralization also reduces systemic risk using the graph model from section 4 and simulated financial systems.

We consider regime (1) as our baseline scenario and will compute all relative impacts with reference to (1).

### 5.2. Simulation Technology

We use a systemic risk engine, see O'Halloran et al. (2017b), to compare the collateralization regimes described above. The engine generates trade relation graphs using the Python libraries numpy.random and networkx and then computes the risk metrics associated to all trades in all trade relations using an open source risk engine, see Open Source Risk Engine (2016). The resulting risk data is then aggregated using pandas. This process is repeated for each of the collateralization regimes such that their effect on the computed risk metrics can be systematically studied.

### 5.3. Synthetic Data

**Figure 7:**
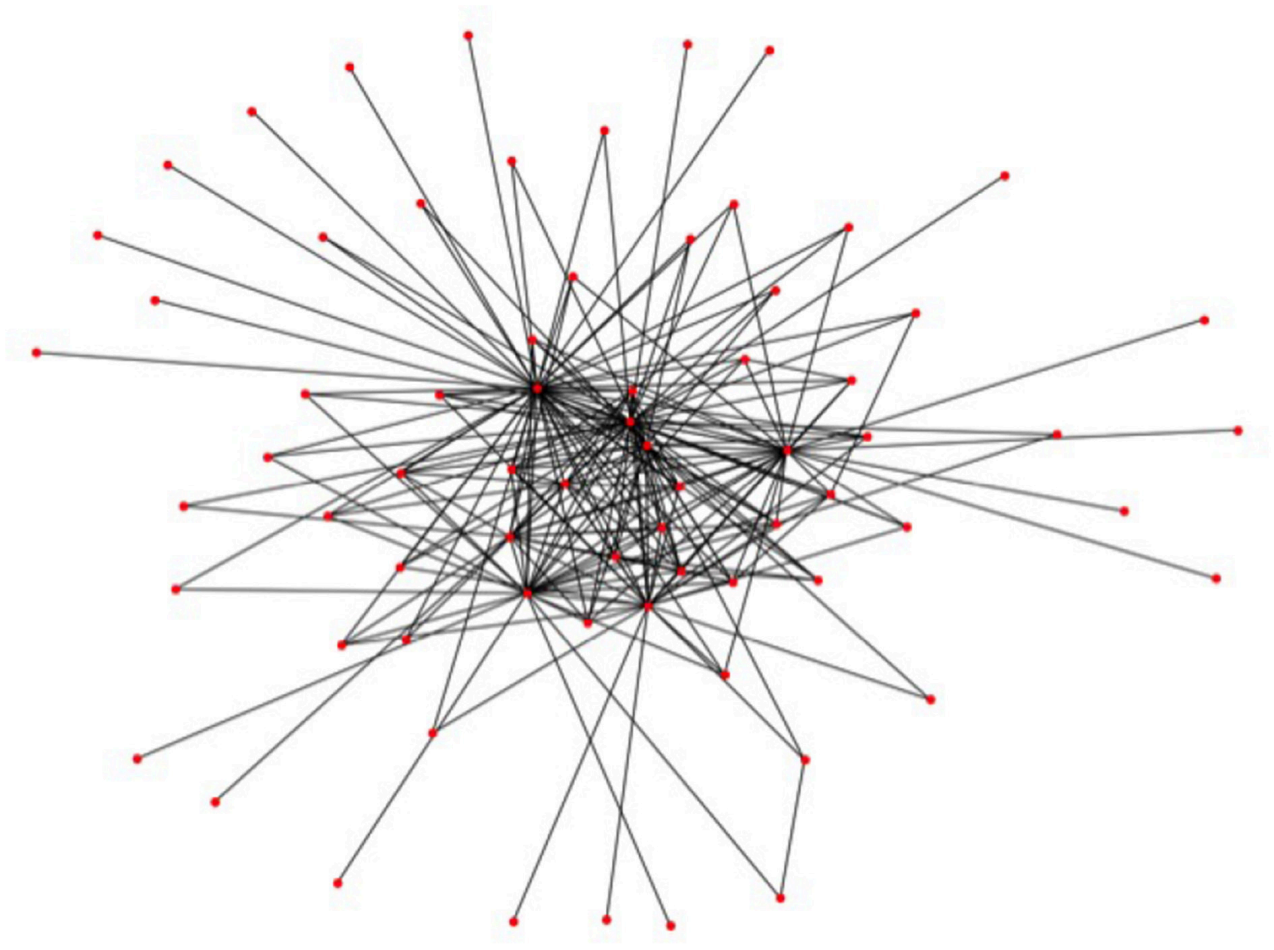
A randomly generated trade relation graph (Reprinted with permission by Columbia University Press).

The first step in the generation of the data is the generation of financial systems like Figure 3, where we want to calibrate the distributions of our random generator to realistic data. A statistical analysis of the macro exposures in the Brazilian banking system carried out in Cont et al. (2013) (based on central bank data) has shown that the degrees of the nodes in the trade relation graph, i.e., the number of links attached to each node, follow approximately a Pareto distribution. Therefore, we randomly generate Pareto distributed sequences and then compute a graph, which realizes that sequence. While the first step is straightforward, the second is a hard problem in discrete mathematics, which is still under active research. For the purposes of this paper, we use the so called erased configuration model as implemented in the Python library networkx and described in Newman (2003). Further details can also be found in Britton et al. (2006), Bayati et al. (2010). The resulting graphs look like Figure 7. We can see that the Pareto distributed node degree yields to graphs which have a few nodes with many links representing a few big banks, and many nodes with only one or a few links representing a large number of smaller firms in the system.

The trades in the trade relations are interest rate swaps (fixed vs. floating) and FX forwards in EUR and USD. Technically, these are implemented as boilerplate ORE XMLs and the trade parameters are chosen at random. For the FX forwards we use uniformly distributed maturities of up to 5Y, uniformly distributed notionals of between 100k and 100m and log-normally distributed strikes. For the interest rate swaps we use the same distributions for the notionals and the fixed rates are uniformly distributed between 0.01 and 5%. A coin flip decides whether or not a generated trade is an FX forward or an interest rate swap and the same applies to the long/short flag.

We run this simulation with parameters, which can be summarized as follows:

- Risk Type: Counterparty Credit Risk
- Risk Metric (choice of weight function *w*): EEPE (Effectivized Expected Positive Exposure)
- Asset classes: IR/FX Derivatives
- Number of financial systems: 10
- Number of banks in each system: ≤50
- Number of trades: 2, 360
- Number of netting sets: 1, 378
- Number of Monte Carlo paths: 500

### 5.4. Results

**Figure 8:**
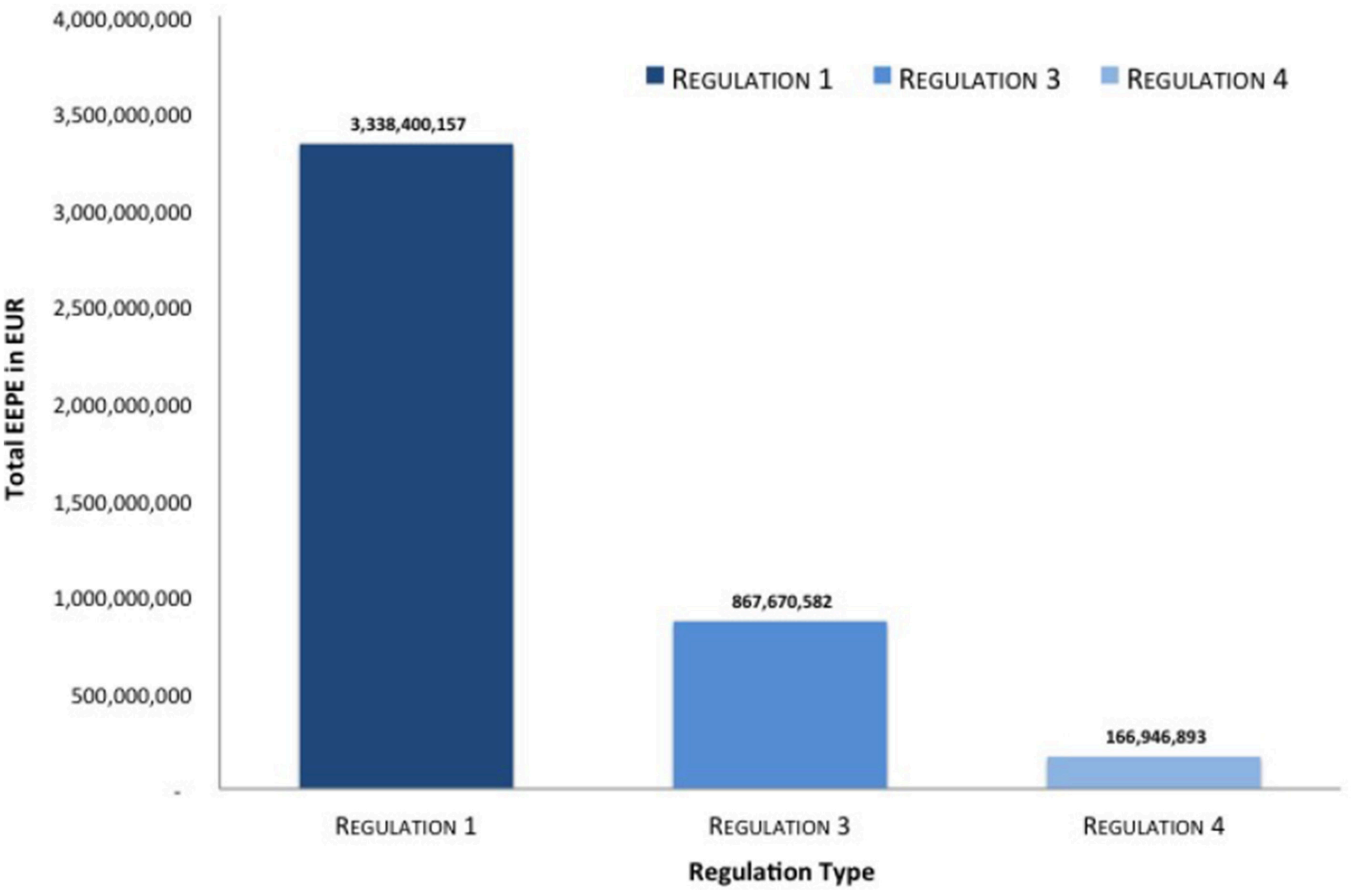
Total reduction of EEPE (Reprinted with permission by Columbia University Press).

In Figure 8 we see a highly aggregated overview of the results of the simulation. We can see that measured in average total levels of credit risk [i.e., *w*(*G*)] measured in *w* = EEPE collateralization reduces this risk. The relative reduction between regime (1), that is the uncollateralized business, and regime (3), that is the fully VM collateralized business, is 74% and the relative reduction between regime (1) and (4), that is the fully VM and IM collateralized business is even 95%. Notice that this level of aggregation is even higher than in macro-prudential regulation as we aggregate across multiple financial systems representing possible future states of the world.

**Figure 9:**
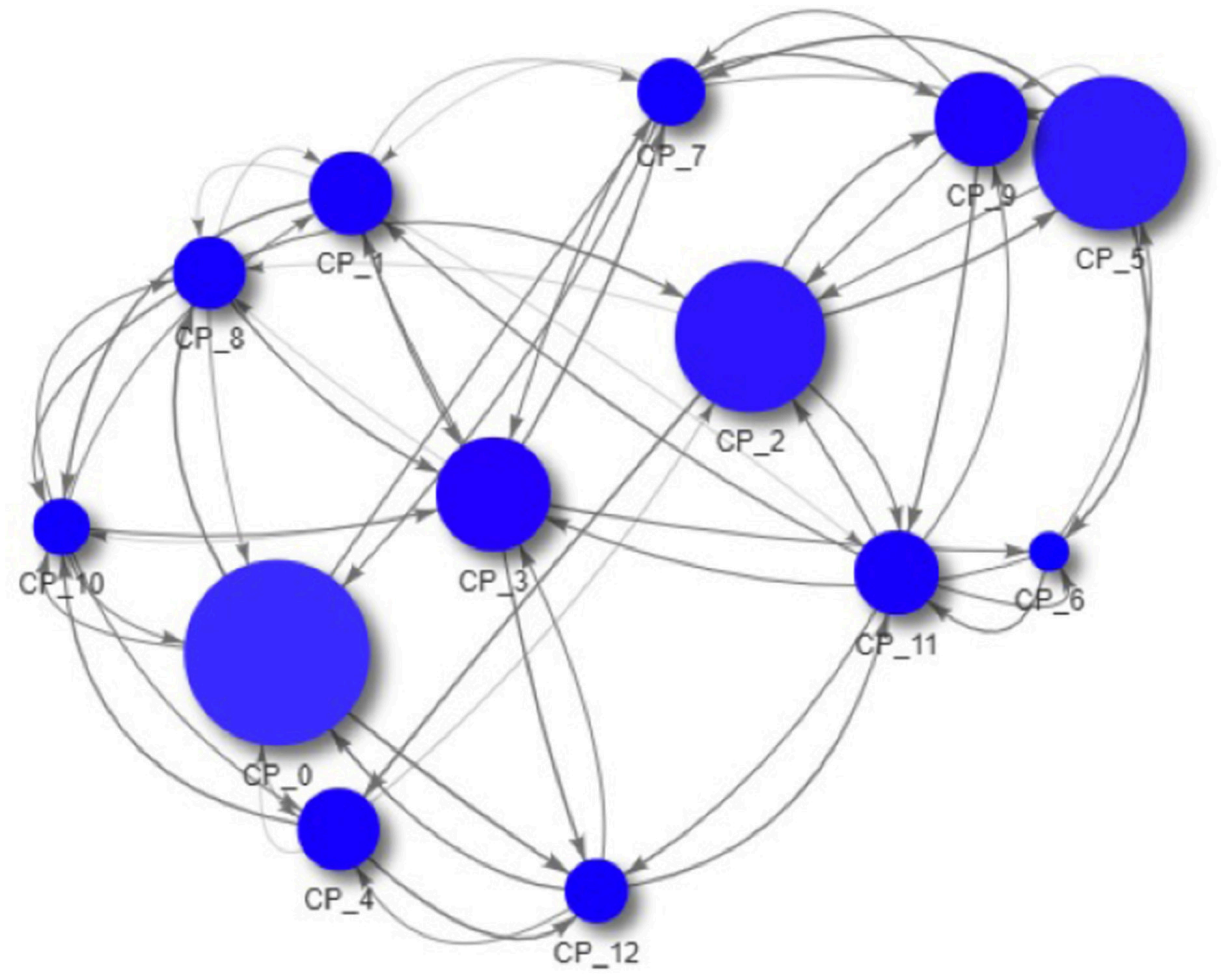
Example of a financial system (uncollateralized) (Reprinted with permission by Columbia University Press).

**Figure 10:**
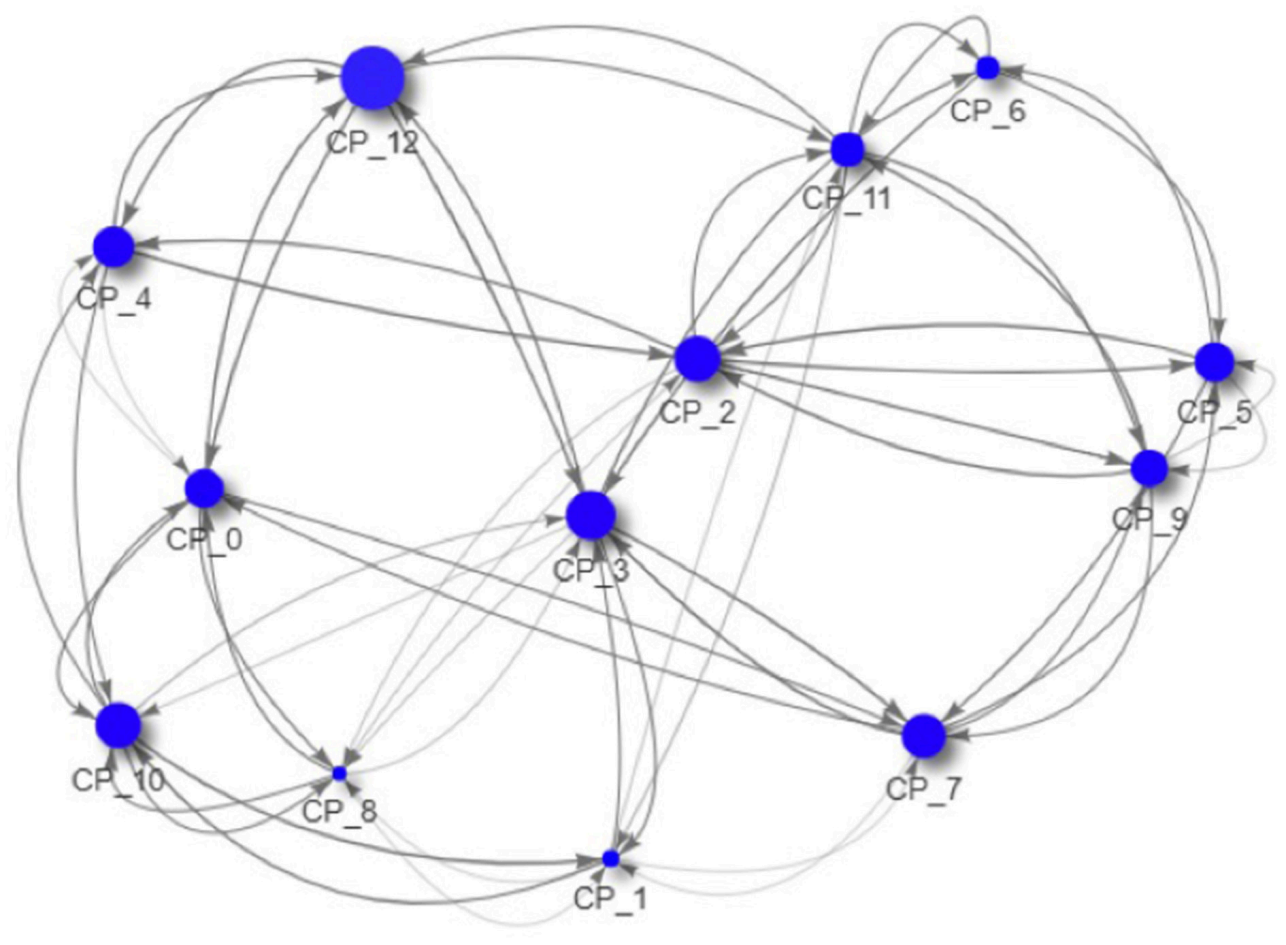
Example of a financial system (VM collateralized) (Reprinted with permission by Columbia University Press).

**Figure 11:**
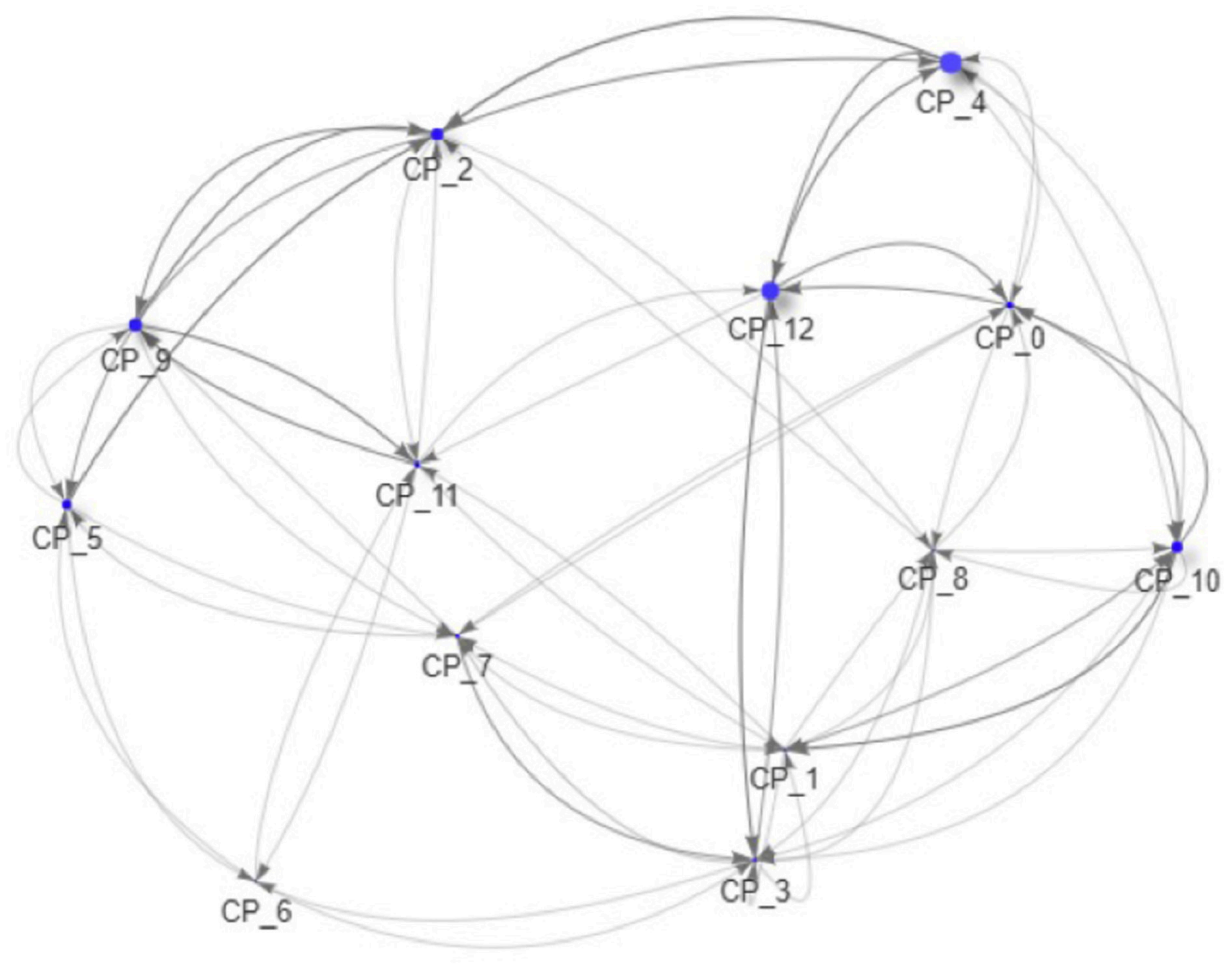
Example of a financial system (VM & IM collateralized) (Reprinted with permission by Columbia University Press).

As all data is created during the simulation and thus completely accessible, we can now drill down to the macro-prudential view and study the impact of those regulations on an example system. In Figures 9–11 we see the risk graph of a financial system under the three regulatory regimes. The size of the node indicates the amount of risk the bank at that node induces into the system, that is the *w*^−^(*b*). We see that collateralization significantly reduces risk in the entire system.

**Figure 12:**
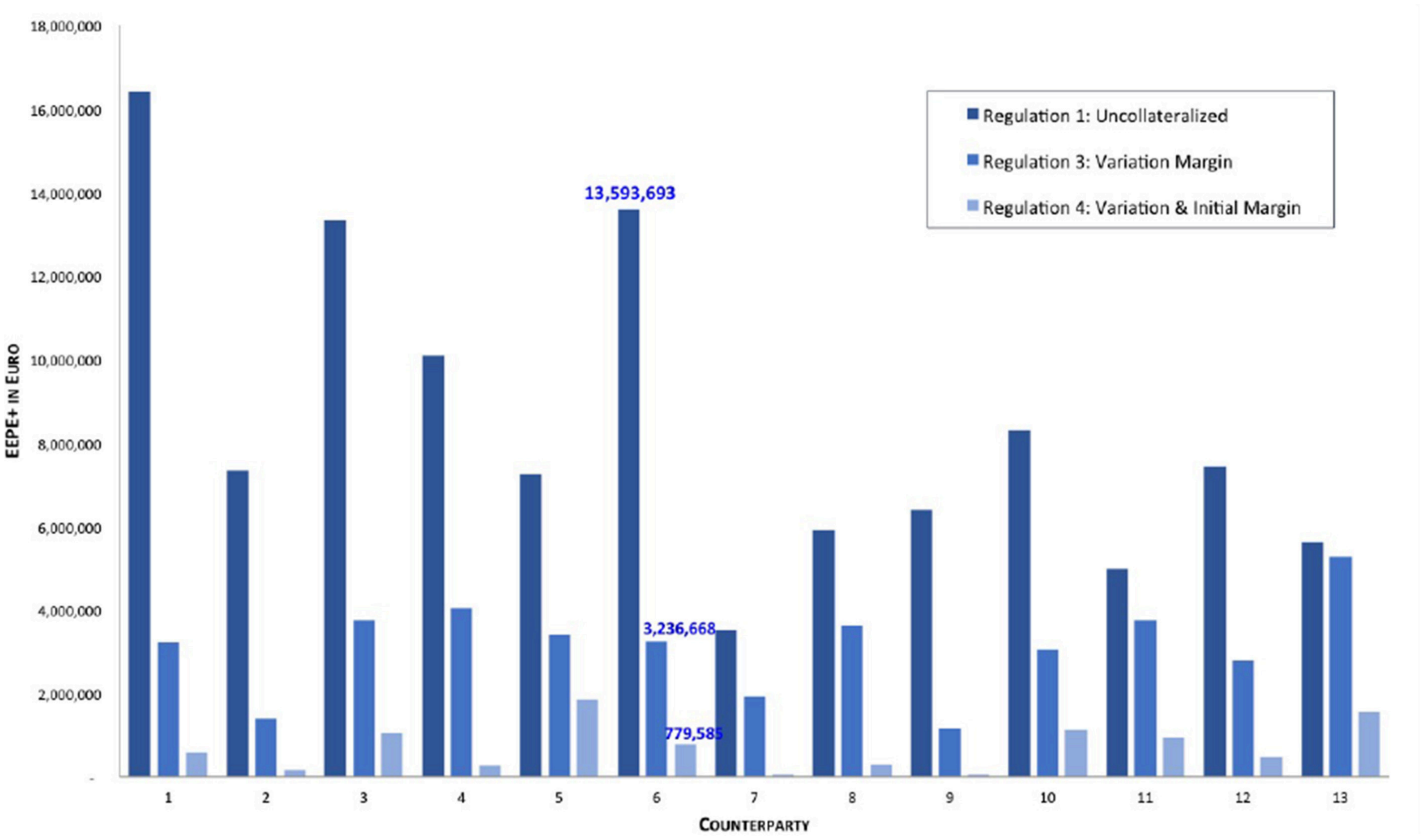
Impact of collateralization on individual banks (EEPE+) (Reprinted with permission by Columbia University Press).

**Figure 13:**
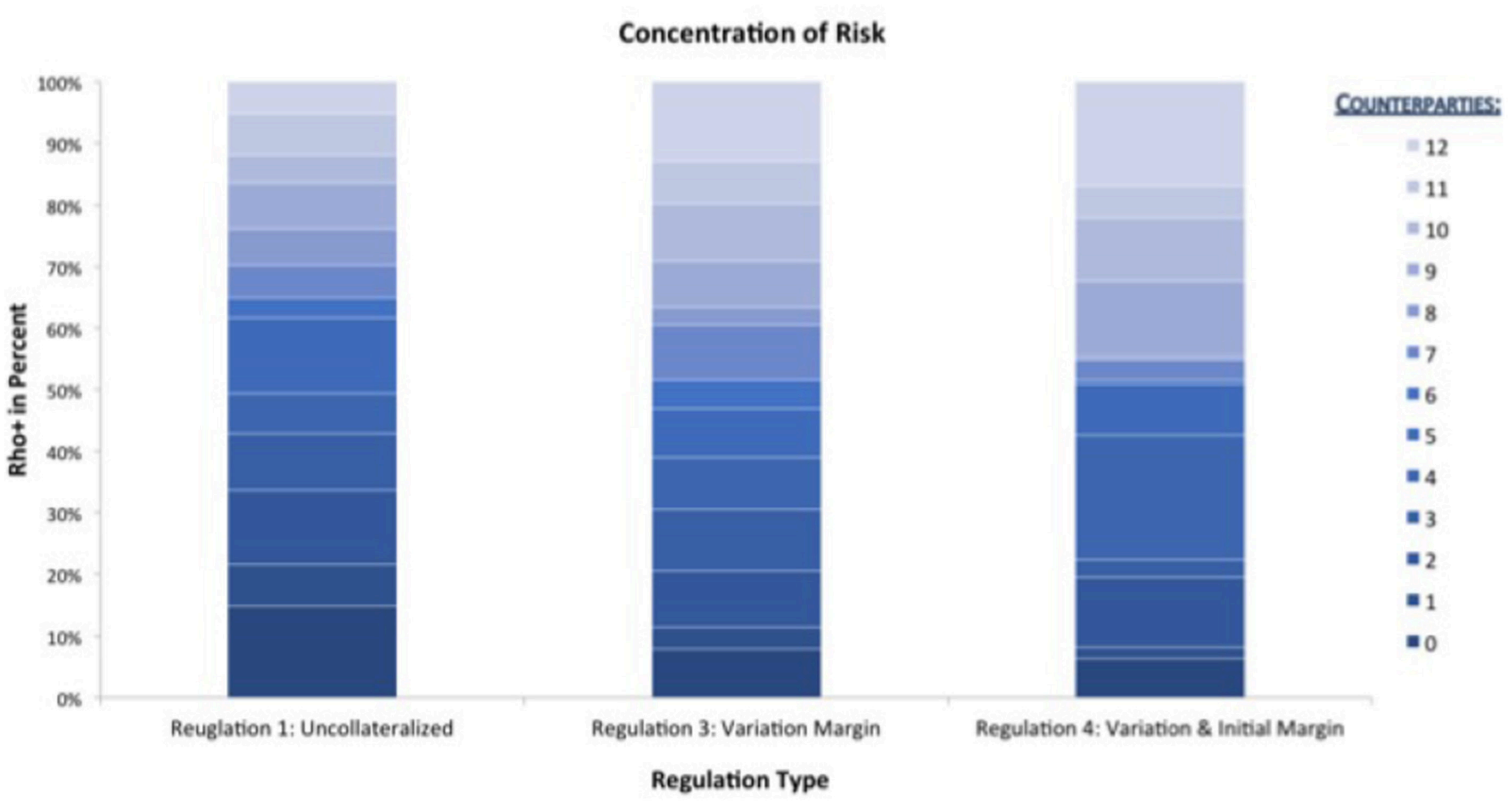
Impact of collateralization on individual banks (ρ+) (Reprinted with permission by Columbia University Press).

This optical impression can be confirmed by drilling down further to the micro-prudential view. In Figure 12 we plot the EEPE+(*b*) for every bank *b* in the system. We can confirm that the impact of collateralization on every bank is qualitatively the same as on the average, that is it reduces individual risk, but the amount of reduction can vary among the banks. It is interesting to note that the concentration of those risks, see Figure 13, i.e., the ρ^+^(*b*) stays mostly the same across the regulations and for banks, where it does change, it is not necessarily smaller. We conclude that collateralization has the desired effect of reducing total levels of risk of each counterparty, but is inadequate to address concentration risks.

**Figure 14:**
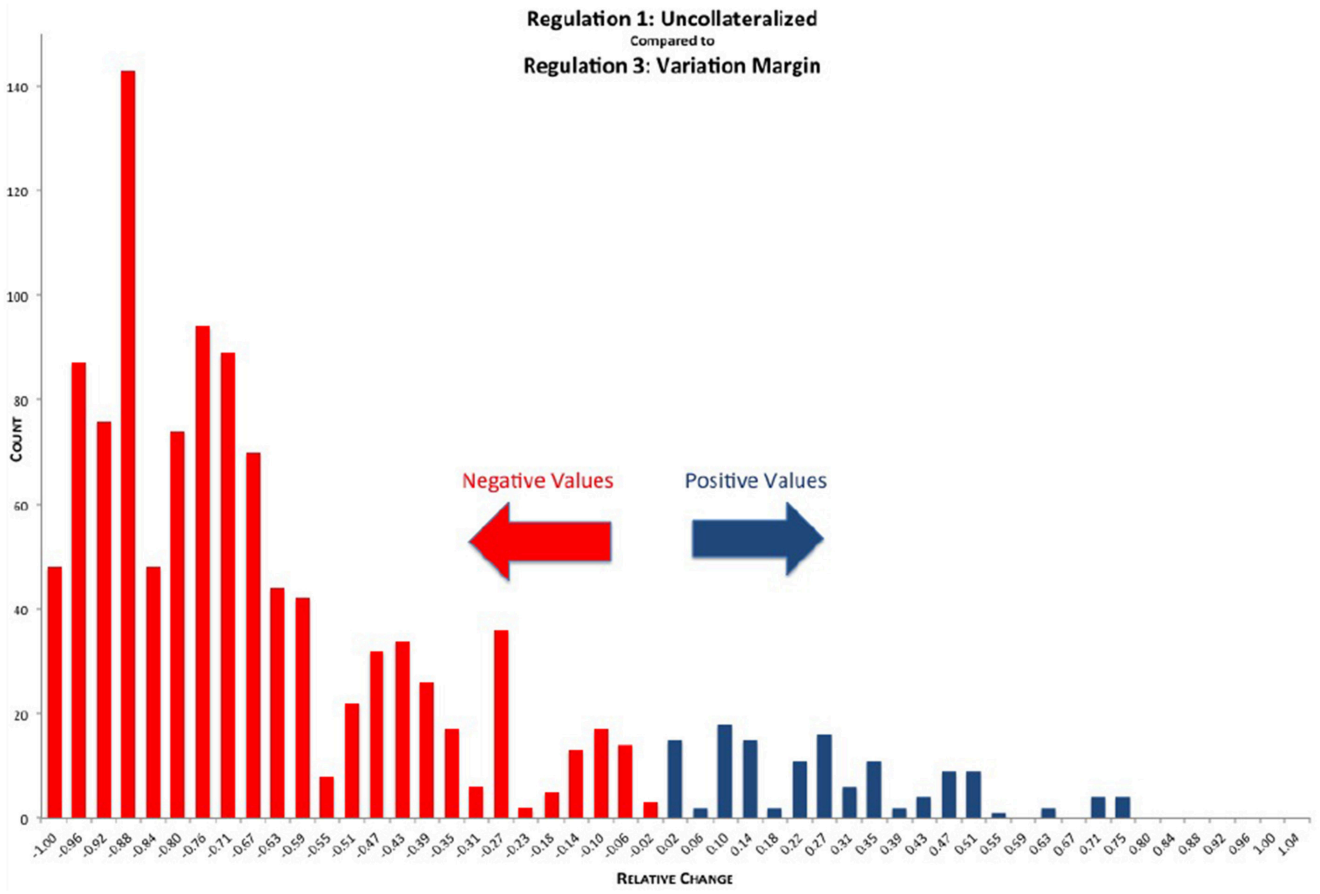
Histogram of relative reduction in EEPE over all netting sets (197 out of 1,378 have more than 150% increase and are not shown, 29 of those have zero uncollateralized EEPE). Mean: −57.42%, SD: 38.33% (Reprinted with permission by Columbia University Press).

We can now drill down even further than the micro-prudential level. As a byproduct of the simulation, we obtain exposure data of 1,378 netting sets, which we can mine to gain insight into all the micro impacts of the various regulations. In Figure 14 we see the distribution of relative reductions in EEPE of the various netting sets when comparing REG_1 (uncollateralized) with REG_3 (VM collateralized). While most of the netting sets show a significant relative reduction in exposure, we can see that some of them also show a significant relative increase in exposure. The explanation for this is as follows: Assume bank *A* has trades in a netting set with bank *B*. These trades are deeply out of the money for bank *A*, meaning the markets have moved into bank *B*'s favor. Then the uncollateralized exposure for bank *A* is very low^6^. Under VM collateralization however, as the trades are deeply in the money for bank *B*, bank *B* will call bank *A* for variation margin. Bank *A* will then pay the variation margin to bank *B*, where it is exposed to the default risk of *B*, because *B* might rehypothecate^7^ this variation margin. In some situations this results in higher exposure under VM collateralization than under no collateralization. We see that on a micro level, VM collateralization can have an adverse effect in rare cases of netting sets, which are deeply out of the money.

**Figure 15:**
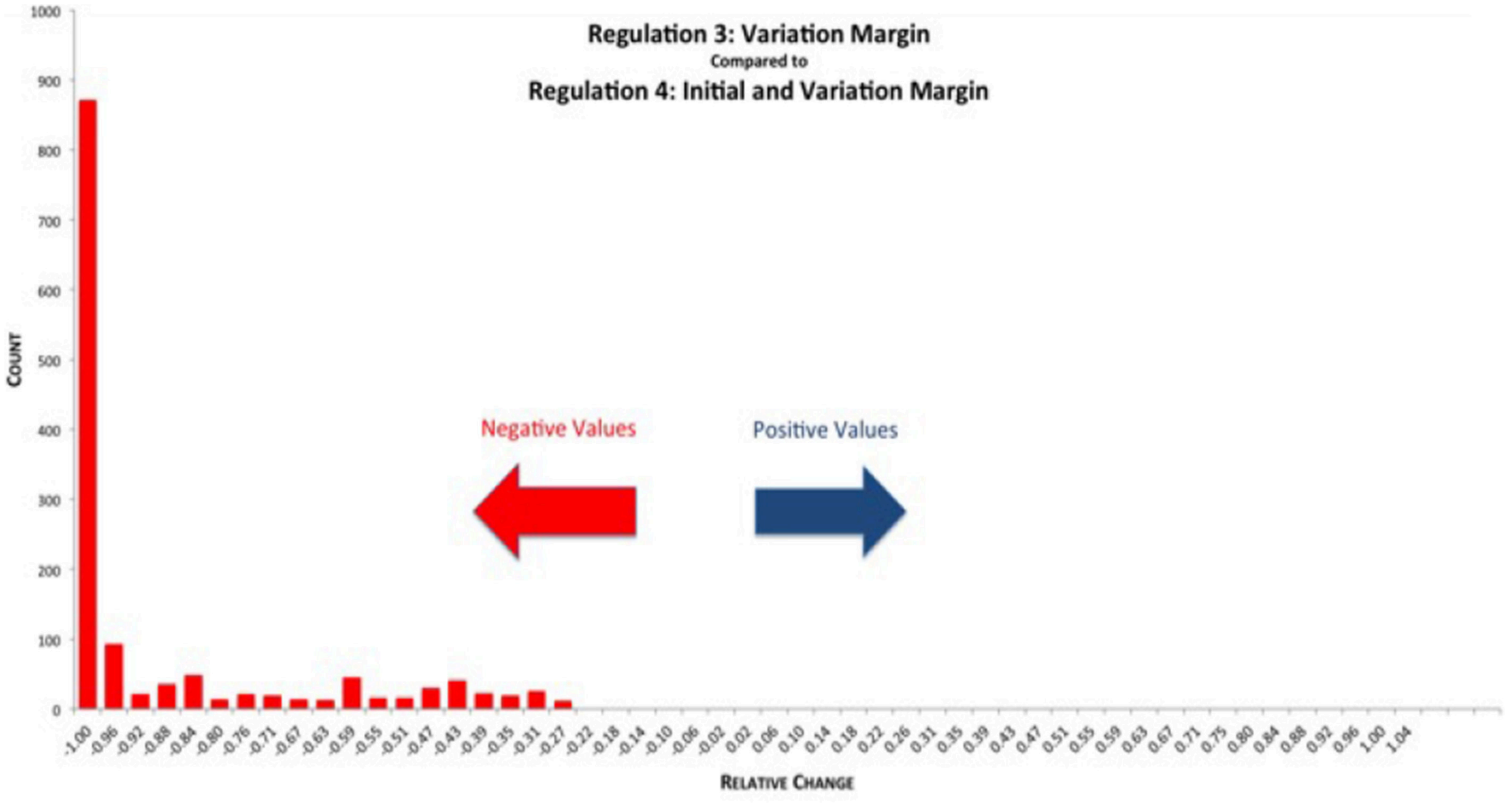
Histogram of relative reduction in EEPE over all netting sets (0 out of 1,378 have more than 150% increase and are not shown, 0 of those have zero VM collateralized EEPE). Mean: −85.78%, SD: 20.76% (Reprinted with permission by Columbia University Press).

Initial Margin cannot be rehypothecated and, therefore, posted Initial Margin is not treated as being at risk^8^. In Figure 15 we see the relative reductions in EEPE of the various netting sets when comparing REG_3 (VM collateralization) vs. REG_4 (VM & IM collateralization). Here, we can see that the effect of the additional IM overcollateralization unambiguously reduces the exposure further.

**Figure 16:**
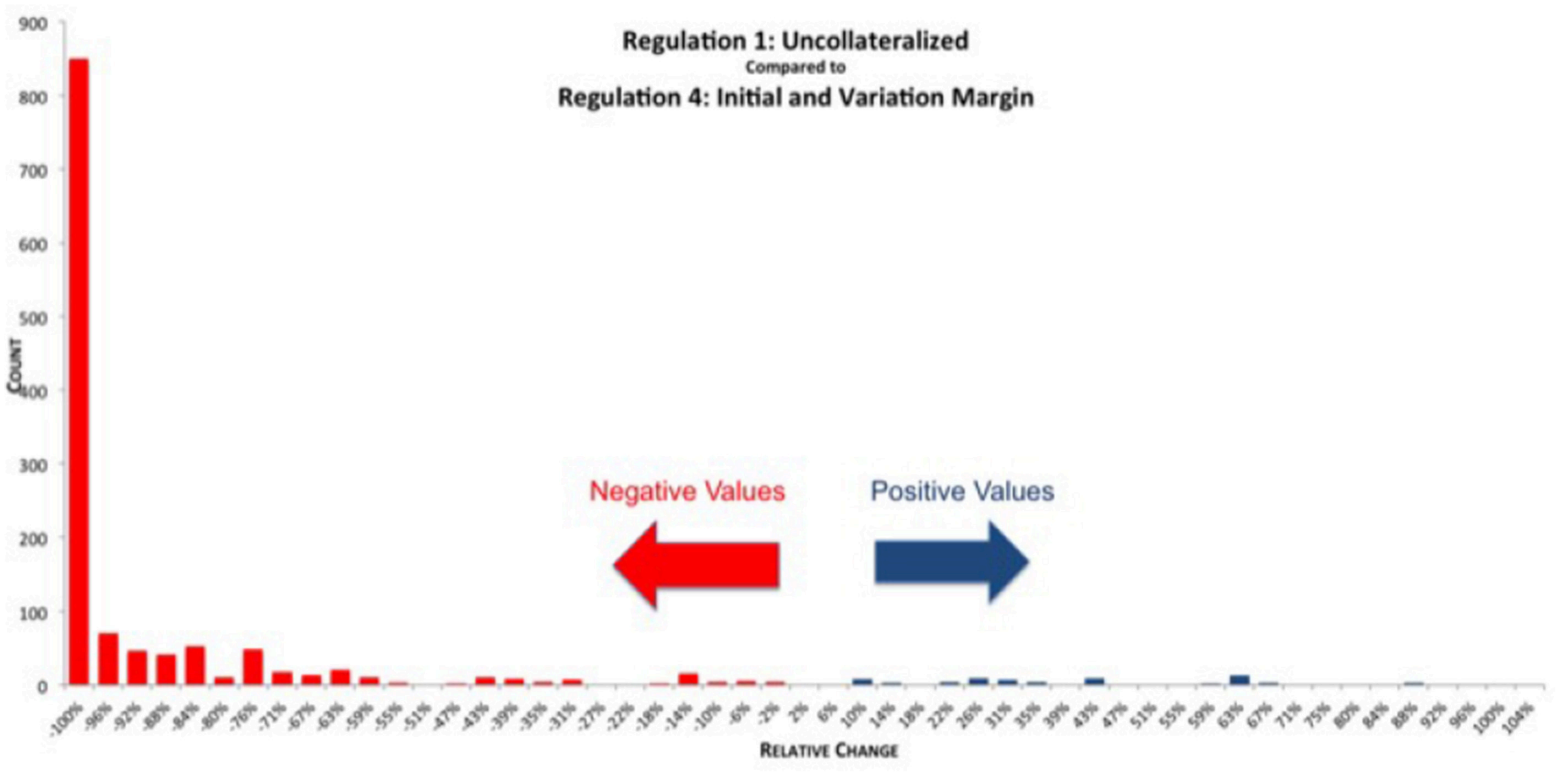
Histogram of relative reduction in EEPE over all netting sets (69 out of 1,378 have more than 150% increase and are not shown, 29 of those have zero uncollateralized EEPE). Mean: −83.83%, SD: 34.44% (Reprinted with permission by Columbia University Press).

When comparing REG_1 (uncollateralized) vs. REG_4 (VM & IM collateralization) directly, we can see in Figure 16 that the reduction in exposure is larger and distributed more narrowly compared with just the VM collateralization, see Figure 14. There are still some netting sets left, which show an increase due to posted variation margin. However, this increase is smaller than under REG_3, as it is partially mitigated by the additional IM collateral.

**Figure 17:**
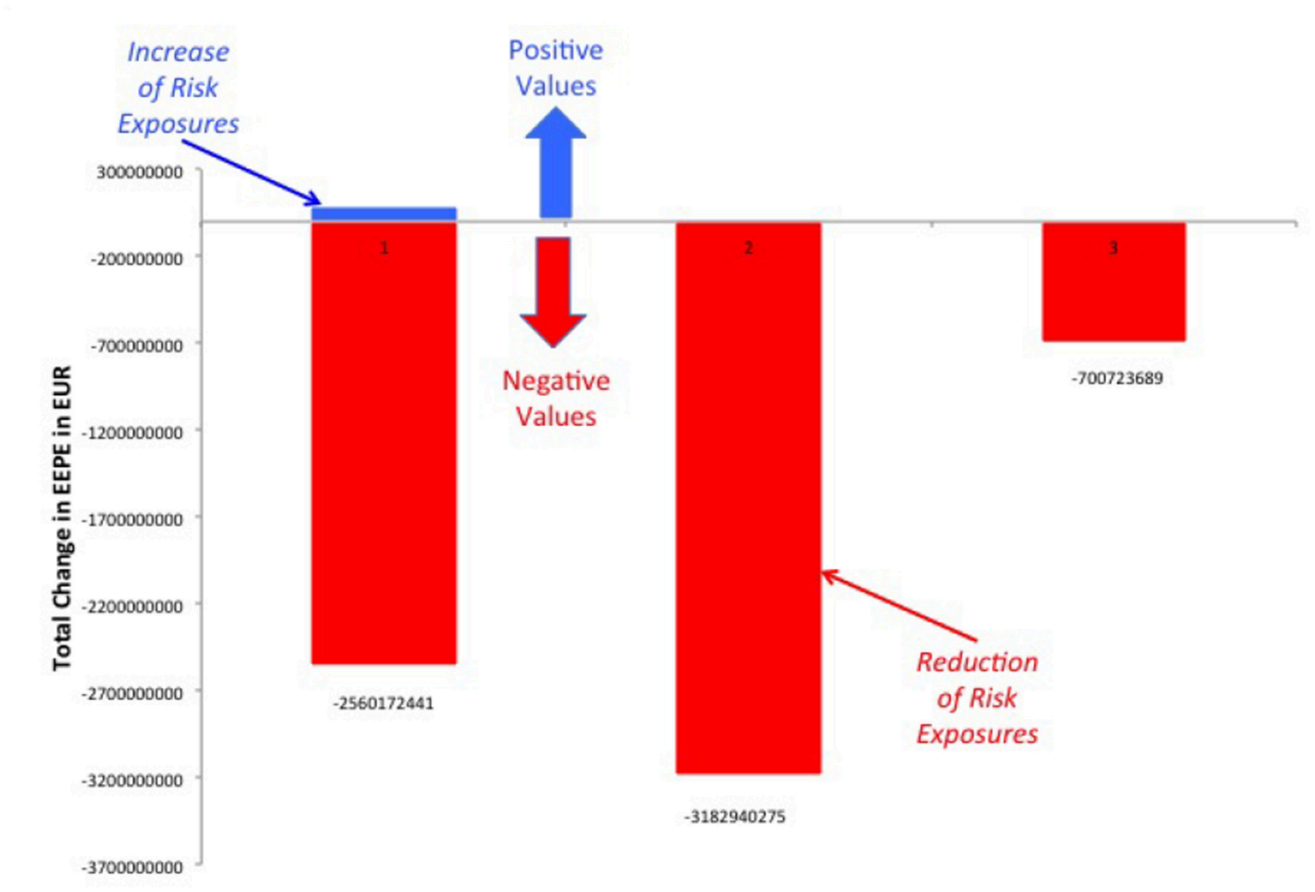
Total increases and decreases in EEPE of all netting sets between the various regimes (Reprinted with permission by Columbia University Press).

It should be noted that while the increases in exposure we see in Figures 14, 16 are large in relative terms, they are actually quite small in absolute terms. In Figure 17 we compute the total increases and decreases in EEPE of all the netting sets separately.

### 5.5. Summary

The directed weighted graph metrics provide a useful comparative statistics to evaluate the impact of various regulatory regimes on systemic risk. Applied to our hypothesis testing we arrive at the following conclusions:

- Collateralization reduces systemic credit risk significantly (measured in EEPE, i.e., the cost of resolving a failed system).
- Collateralization does not materially change the concentration of credit risk in a financial system.
- In corner cases (deeply out of the money portfolios), VM collateralization can increase credit risk.

Notice that these results are an interplay of the aggregated macro-exposures and a systematic analysis of all micro-exposures, which would not be possible outside of the present framework.

## 6. Conclusion

Over the past two decades, the interconnected nature of global financial markets has increased dramatically, exacerbating threats to the financial system through the domino effect, the fire-sale effect, and oversized role certain firms. Just like predicting the weather, financial service firms are now more interconnected and inherently more complex than ever before. The financial crisis highlighted the dangers of relying too heavily on proprietary models developed in silos. The open source paradigm introduced provides a means to benchmark models and to have common standards across the industry. The analytic approach adopted merges the structural and predictive properties of graph model and AI techniques to generate a financial system from real distributions of bank trading data.

Our analysis advances the literature in three ways:

1. Provides a simulation environment that enables iterative stress testing to decipher patterns and linkages in the data, thereby providing more robust models and estimates of systemic risk;
2. Generates entire financial systems from a single trade to the entire trade relation graph, resulting in a graph model of systemic risk derived from random distributions of bank data; and
3. Aggregates standardized micro-prudential risk exposures into a macro-systemic risk metric that captures both the amount of risk a firm generates and the amount it absorbs.

We will expand the substantive analysis and methodological approach developed here in a number of directions:

**Large scale simulation:** The simulation performed to obtain the results in section V-D ran on a standard desktop computer. We plan to deploy the systemic risk engine in a cloud environment and run a large scale simulation to achieve an even higher statistical robustness.
**Dependence on distributions of the trades:** During the random generation of the trade relations, various distributional choices have to be made. It is interesting to study the dependence of the results on those choices. We expect them to be fairly stable under distributions.
**Agent based creation of trade relation graphs:** The current model assumes a Pareto distribution of the trade relations. It would be interesting to enhance the nodes representing the banks in the graph model with dynamic agent based rules of trading and study under what conditions the resulting trade relations are Pareto distributed.
**Joint modeling of all major risk classes:** In the present article we study the impact of collateralization on credit risk. However, regulation can affect all types of risk and the metrics used to measure it. We plan to conduct a joint analysis of market risk, credit risk, liquidity risk, operational risk and model risk. In fact, there is significant interplay between the various risks. Figures 5, 6 highlight a paradox: the notional amount of OTC derivatives increased simultaneously as market risk decreased and credit risk remained changed. One explanation is that collateralization may decrease market risk at the expense of increased liquidity risk. We can test this possibility with the AI framework detailed above^9^.
**Initial Margin and Funding Costs:** We believe that the key to understanding the interplay between credit risk and liquidity risk, in particular when studying the impact of collateralization, is its effect on funding costs and other value adjustments of derivative trades, the so called XVAs. These quantify the price of the reduction in risk.
**Derivatives Market vs. Money Market:** It is to be expected that collateralization will not only impact the derivatives market, but also the money market. As initial margin cannot be rehypothecated, its impact could be large. It is therefore interesting to study the interplay between those markets, both in case studies and simulations.
**Central Clearing:** The current analysis focuses on the study of the impact of collateralization on systemic risk as this was one of the major regulatory responses to the crisis. Another response was the incentivization of central clearing, which can be studied in a similar fashion. Notice that the graph model presented in section 4 is already able to capture the effect of this regulation: Any bilateral trade relation of a bank *A* with a bank *B* has to be replaced by two trade relations—one of bank *A* with the clearing house and another one for bank *B* with the clearing house. We expect to obtain results quantifying how much safer a clearing house needs to be in order to reduce systemic risk compared to bilateral trading.

## Author Contributions

All authors listed have made a substantial, direct and intellectual contribution to the work, and approved it for publication.

## Conflict of Interest Statement

The authors declare that the research was conducted in the absence of any commercial or financial relationships that could be construed as a potential conflict of interest.

## Footnotes

^1^See Kamani et al. (2018) for an application of this technique to forecast severe climate events.

^2^Similarly, in a meta analysis of the literature on systemic financial risk, Silva et al. (2017) find that from a sample of 266 articles published from 1990 to 2016, 134 articles directly addressed measures or indices of systemic risk.

^3^This is a regulatory standard metric to measure exposure. Notice that the exposure is a key ingredient in the calculation of capital requirements. Thus, a reduction in exposure automatically causes a reduction in capital requirements.

^4^See Bank of International Settlements, Statistical release: OTC derivatives statistics at end- June 2018.

^5^In the language of section 4 this means that collateralization reduces the *w*^−(*b*)^, i.e., the amount of risk bank is exposed to, where *w* is a credit risk metric (EEPE in our case).

^6^Due to the finite number of MonteCarlo paths, it is sometimes even numerically zero in the simulation.

^7^i.e., posting margin received from one counterparty to another.

^8^It should be highlighted that in our simulation we model the bilateral trading between various banks, where Initial Margin is posted into segregated accounts. Derivatives that are cleared through a central counterparty (CCP) or exchange traded derivatives (ETDs) are not in scope of this simulation.

^9^Another way the divergence between market and credit risk can occur is if banks resort to means outside the regulatory matrix. For example, banks can transact derivative contracts in tax havens, use instruments currently not covered by regulations or decide not to insure against the possibility of default altogether.

## References

[B1] Adrian T. Brunnermeier M. K. (2016). CoVaR. Am. Econ. Rev. 106, 1705–1741. 10.1257/aer.20120555

[B2] Andersen L. Pykhtin M. Sokol A. (2016). Credit Exposure in the Presence of Initial Margin. SSRN pre-print. Available online at: https://ssrn.com/abstract=2806156

[B3] Andersen L. Pykhtin M. Sokol A. (2017). Does Initial Marign Eliminate Counterparty Risk? Risk Magazine.

[B4] Anfuso F. Aziz D. Giltinan P. Loukopoulos K. (2017). A Sound Modelling and Backtesting Framework for Forecasting Initial Margin Requirements. 86–91.

[B5] Bales M. Johnson S. (2006). Graph theoretic modeling of large-scale semantic networks. J. Biomed. Informatics 39, 451–464. 10.1016/j.jbi.2005.10.007 16442849

[B6] Basel Committee on Banking Supervision (2015). Margin Requirements for Non-centrally Cleared Derivatives. BCBS 317. Available online at: https://www.bis.org/bcbs/publ/d317.htm (accessed February 3, 2019).

[B7] Battiston S. Gatti D. Gallegati M. Greenwald B. Stiglitz J. (2012b). Default cascades: When does risk diversification increase stability? J. Financ. Stabil. 8, 138–149. 10.1016/j.jfs.2012.01.002

[B8] Battiston S. Gatti D. D. Gallegati M. Greenwald B. Stiglitz J. (2012a). Liaisons dangereuses: increasing connectivity, risk sharing, and systemic risk. J. Dyn. Control 36, 1121–1141. 10.1016/j.jedc.2012.04.001

[B9] Battiston S. Martinez-Jaramillo S. (2018). Financial networks and stress testing: Challenges and new research avenues for systemic risk analysis and financial stability implications. J. Financ. Stabil. 35, 6–16. 10.1016/j.jfs.2018.03.010

[B10] Bayati M. Kim J. Saberi A. (2010). A sequential algorithm for generating random graphs. Algorithmica 58, 860–910. 10.1007/s00453-009-9340-1

[B11] Billio M. Getmansky M. Lo A. W. Pelizzon L. (2012). Econometric measures of connectedness and systemic risk in the finance and insurance sectors. J. Financ. Econ. 104, 535–559. 10.1016/j.jfineco.2011.12.010

[B12] Bisias D. Flood M. Lo A. W. Stavros V. (2012). A Survey of Systemic Risk Analytics. Washington, DC: Office of Financial Research, Working Paper #0001.

[B13] Britton T. Deijfen M. Martin-Löf A. (2006). Generating simple random graphs with prescribed degree distribution. J. Stat. Phys. 124, 1377–1397. 10.1007/s10955-006-9168-x

[B14] Caccioli F. Farmer J. D. Foti N. Rockmore D. (2015). Overlapping portfolios, contagion, and financial stability. J. Econ. Dyn. Control 51, 50–63. 10.1016/j.jedc.2014.09.041

[B15] Capponi A. Chen P. (2015). Systemic risk mitigation in financial networks. J. Dyn. Control 58, 152–166. 10.1016/j.jedc.2015.06.008

[B16] Caspers P. Giltinan P. Lichters R. Nowaczyk N. (2017). Forecasting initial margin requirements–a model evaluation. J. Risk Manage. Financ. Inst. 10, 365–394. 10.2139/ssrn.2911167

[B17] Columbia University FinTech Lab (2018). Available online at: http://fintech.datascience.columbia.edu/

[B18] Cont R. Moussa A. Santos E. (2013). Network structure and systemic risk in banking systems, in Handbook on Systemic Risk, eds Fouque J. Langsam J. (Cambridge: Cambridge University Press), 327–368.

[B19] Detering N. Meyer-Brandis T. Panagiotou K. Ritter D. (2016). Managing default contagion in inhomogeneous financial networks. arXiv[Preprint]. arXiv:1610.09542.

[B20] Erdős P. Rényi A. (1959). On random graphs. Publ. Math. 6, 290–297.

[B21] Erdős P. Rényi A. (1960). On the evolution of random graphs. Publ. Math. Inst. Hung. Acad. Sci. 5, 17–61.

[B22] ISDA (2016). ISDA SIMM (TM) Methodology. Version R1.0, September 2016.

[B23] Kamani M. M. Farhat F. Wistar S. Wang J. Z. (2018). Skeleton matching with applications in severe weather detection. Appl. Soft Comput. 70, 1154–1166. 10.1016/j.asoc.2017.05.037

[B24] Lichters R. Stamm R. Gallagher D. (2015). Modern Derivatives Pricing and Credit Exposure Analysis: Theory and Practice of CVA and XVA Pricing, Exposure Simulation and Backtesting (Applied Quantitative Finance). London: Palgrave Macmillan.

[B25] Newman M. E. J. (2003). The structure and function of complex networks. SIAM Rev. 45, 167–256. 10.1137/S003614450342480

[B26] O'Halloran S. Nowaczyk N. Gallagher D. (2017a). Big data and graph theoretic models: simulating the impact of collateralization on a financial system, in Proceedings of the 2017 IEEE/ACM International Conference on Advances in Social Networks Analysis and Mining 2017, ASONAM '17 (New York, NY: ACM), 1056–1064. 10.1145/3110025.3120989

[B27] O'Halloran S. Nowaczyk N. Gallagher D. (2017b). A data science approach to predict the impact of collateralization on systemic risk. in After the Crash: Financial Crises and Regulatory Responses, eds O'Halloran S. Groll T. (New York, NY: Columbia University Press).

[B28] Open Source Risk Engine User Guide (2017). Available online at: http://www.opensourcerisk.org/documentation/

[B29] Open Source Risk Engine (2016). Available online at: www.opensourcerisk.org. First release in October.

[B30] Sedunov J. (2016). What is the systemic risk exposure of financial institutions? J. Financ. Stabil. 24, 71–87. 10.1016/j.jfs.2016.04.005

[B31] Silva W. Kimura H. Sobreiro V. (2017). An analysis of the literature on systemic financial risk: a survey. J. Financ. Stabil. 28, 91–114. 10.1016/j.jfs.2016.12.004

